# A community-wide school health project for the promotion of smoke-free homes

**DOI:** 10.1186/s13104-015-1555-4

**Published:** 2015-11-26

**Authors:** Alice Yuen Loke, Y. W. Mak

**Affiliations:** Family and Community Health Research, School of Nursing, The Hong Kong Polytechnic University, Hung Hom, Hong Kong

**Keywords:** Smoke-free home, School health promotion, Community project

## Abstract

**Background:**

A community-wide school health project for the promotion of smoke-free homes was launched in June 2010 with the aim of promoting the benefits of smoke-free homes to all school-aged children (aged 6–18), and indirectly to their parents and family members. The 1-year project included health talks on a smoke-free life; the distribution of educational leaflets; slogan and visual art competitions; and a health fair held in June 2011. Two sets of questionnaires were developed to solicit a resolution and action from the participants regarding the establishment of a smoke-free home, and their decision to stay smoke-free. This is a paper to report on the activities of this project, the attempts to reach out to school-aged children, and their indications of agreement with, support for, and commitment to promoting smoke-free homes.

**Results:**

The project reached an estimated 12,800 school-aged children in Hong Kong. A large proportion of those received educational leaflets (69.6–88.2 %). Of those who participated in the health fair, 69.7–87.6 % agreed to promote the concept of smoke-free homes to friends and family. More primary than secondary students pledged to not take up smoking (90.8 vs 85.8 %). About 82 % of those who had experimented with smoking pledged to stop. A small proportion of them reported already having established a smoke-free policy at home (14.9 %), placed a ‘No Smoking’ sign at home (16.4 %), informed visitors of their smoke-free policy at home (12.9 %), and asked visitors to dispose of lit cigarettes before entering their home (15.9 %).

**Discussion:**

This community-wide school health project on the benefits of smoke-free homes reached a large number of students, and indirectly to family members, and home visitors. Public health efforts of this kind should be continued to reach younger generations and the general public.

**Electronic supplementary material:**

The online version of this article (doi:10.1186/s13104-015-1555-4) contains supplementary material, which is available to authorized users.

## Background

In 2007, Hong Kong passed legislation banning smoking in indoor public places and workplaces, including restaurants, offices, schools, hospitals, markets, and bars [1]. A report in Canada suggested that besides offering practical protection against passive smoking, smoking bans also decreased the normative status of smoking, and may discourage people from taking up smoking as well as encourage smokers to quit smoking [2]. In China, the ban on smoking that was put in place in 2011 generated increases in online searches for ‘electronic cigarettes’ and ‘quit smoking’ [3]. In the United States, it was estimated in the 1990s that workplace smoking bans had led to a 5 % reduction in the prevalence of smoking, and to a 10 % reduction in the daily consumption of cigarettes [4]. It was reported in a systematic review that the establishment of a policy on smoke-free workplaces was effective in changing the smoking behaviours of smokers at work, and thus in protecting non-smoking co-workers from the dangers of passive smoking [5].

The policy to ban smoking in public places was established to protect the general public from exposure to smoke in enclosed public places; however, these regulations have done little to decrease the exposure of children to passive smoking from their parents [6]. In fact, since the establishment of regulations to ban smoking at work and in public places, there has been increasing concern that children are being exposed to second-hand smoke in the home [7].

### Adolescents and smoking

In a report, the Hong Kong Council on Smoking and Health [8] stated that about 27 % of secondary students in years 1–3 (aged 13–15) have tried smoking. Studies have revealed that adolescents are mostly influenced by the smoking habits of their parents and friends [9, 10]. The family unit is the primary source from which social factors that may underlie an individual’s smoking behaviour are transmitted [11]. It has been reported that young children who have experimented with smoking are more likely to have experienced second-hand smoke within their family, and those who associate with friends who smoke are at an increased risk [12].

It has been reported that among those who have ever used cigarettes, having a friend who smokes cigarettes was the key factor in whether an adolescent will pick up a cigarette [13]. Nevertheless, research suggests that adolescents from homes with a total ban of smoking were less likely to have experimented with smoking [14], and this association remains regardless of the smoking habits of their peers. The study showed that after the effects of having friends who smoke were excluded, adolescents from homes that allowed smoking were about 50 % more likely to be smokers (OR = 1.50) than adolescents from homes where smoking was completely banned [14]. This suggests that adopting a ban on smoking at home can reduce the likelihood that adolescents will try tobacco, regardless of whether or not their friends smoke, and also reduce some of the influence that having friends who smoke can have on the smoking behaviour of adolescents.

### Smoke-free homes

An epidemiological analysis of the changing pattern of cigarette consumption in the United States found that both smoke-free workplaces and smoke-free homes were associated with a decrease in the prevalence of smoking and in the average consumption of cigarettes from 1992 to 2002 [15]. With the success of the policy on smoke-free workplaces, there have been widespread suggestions by policy makers and public health professionals in places such as the United States [16], Australia [17], and the United Kingdom [7] that a smoke-free policy be adopted in homes.

A study in Hong Kong reported that about 85.6 % of smoking parents smoked at home [18]. It is speculated that besides protecting children from exposure to second-hand smoke a smoke-free home will also encourage smoking parents to quit or reduce their cigarette consumption.

### Underpinning theoretical model

A school health project on the ‘Promotion of Smoke-Free Homes (SFH)’ was developed based on childhood health promotion models by Bandura [19]. The contention is that it is easier to prevent health risk behaviours among young children than to try to change such behaviours later in life when they became a habit. Although health behaviours are rooted in the practices of families, schools play an important role in promoting the health of young people. Health promotions must be constructed as part of a social commitment, and they are more successful with the concerted efforts of schools, families, and the community [20]. This health promotion project was created using a school-based approach in which the school operates in concert with the home and the community—in other words, the project was operated in the schools but was not of the schools.

### Aim and objectives of the project/study

This school health project on the ‘Promotion of Smoke-Free Homes (SFH)’ was launched with the aim of educating school-aged children, as well as their parents, about the benefits of smoke-free homes as to reduce the children’s exposure to passive smoking, and to normalize the idea of a smoke-free environment among the school-aged children and the general public.

The specific objectives of this health promotion project were to: (1) reach school-aged children to promote the message of smoke-free homes, (2) increase the students’ knowledge and awareness of smoke-free homes, and encourage them to promote the concept to family members and friends by advising them to quit and not to smoke at home, and (3) increase the public’s knowledge of smoking and passive smoking, and their commitment to maintaining an SFH.

This paper reports the outcome of these activities in reaching the school-aged children, and on their indications of agreement with, support for, and commitment to promoting smoke-free homes.

## Methods

This was a survey study using questionnaires. The questionnaires were completed by school-aged children, their parents, and the participants of a school health project on the ‘Promotion of Smoke-Free Homes (SFH)’.

### Setting

In the month of June, 2011, a total of 1103 promotional packages were sent to all mainstream primary (n = 594) and secondary schools (n = 509) listed under the Education Bureau in Hong Kong, inviting them to participate in this project. Special education schools and international schools were excluded. A total of 38 schools responded to our invitation, assisted to distribute the educational leaflets to their students and had their students completed a questionnaire.

The participants of the health fair held on July 2012, were randomly selected at the health fair to complete the questionnaire. About 50 copies were randomly distributed to participants 6 times throughout the health fair, hourly from 11 a.m. to 4 p.m., at the Health Fair.

### Target population

The SFH project targeted all school-aged children studying in all primary and secondary schools (aged 6–18) in Hong Kong. It also indirectly targeted their families, including parents and other family members. The aim of the SFH project was to disseminate the message of “Smoke-free Homes” to families through students in schools, whether or not these families included members who smoked.

### Project activities

‘Promoting Smoke-Free Homes’ (SFH) was a 1-year health promotion project that kicked off in June 2010. It consisted of a series of activities: (1) the provision of health talks in primary and secondary schools to provide students with first-hand knowledge of the benefits of smoke-free homes; (2) the distribution of educational leaflets to students and their families on the benefits of an SFH and how to create a smoke-free home; (3) the launch of a slogan and a visual-arts competition to encourage primary and secondary school students to spread the message on the benefits of living in a smoke-free home; and (4) a health fair and award ceremony with various activities and relevant educational talks relating to establishing ‘Smoke-Free Homes’.

#### Promotional materials of the community-wide school project

A series of promotional materials were created. The promotional poster included: information on the offer of free smoking-related health talks to schools, a call for entries for the Chinese and English slogan, and comic drawing story-telling competitions. A project logo on the poster was designed to catch the attention of children and families, and to give the concept of ‘smoke-free homes’ a clean, healthy, and joyful image, by depicting plants and butterflies around a house. A total of 3000 copies were printed for distribution to primary and secondary schools.

Promotional packages, including a poster, a health education leaflet, an invitation letter to school principals, and a reply proforma were sent to all mainstream primary and secondary schools under the Hong Kong Government’s Education Bureau at the beginning of the school year in early September 2010. The packages served as the first announcement of the project to all schools. Second and third announcements were emailed to all schools, and again faxed to those schools that returned a proforma indicating their support and willingness to take part in SFH activities.

An SFH webpage was created to make the SFH project easily accessible to students, parents, schools, and the public. It included a brief introduction to the SFH project, the purpose of the project, and the target population, a list of the promotional activities, downloadable competition forms for interested participants, and contact information for those interested in making queries.

The Communications and Public Affairs Department of the University also helped to disseminate the project activities to the press, and there were newspaper announcements about the project’s slogan and visual arts competitions. Promotional banners for the 2011 Health Fair were displayed in the university 3 weeks before the event.

#### Educational leaflets for the promotion of smoke-free homes

An SFH educational leaflet was created for the purpose of providing information on the importance of ‘smoke-free homes’ and tips on establishing such homes. It also included information on how exposure to second-hand smoke affects one’s health. The leaflet was designed with colourful and attractive pictures to attract attention. A total of 8000 educational leaflets were printed for distribution to all primary and secondary schools in Hong Kong.

#### Health talk on ‘Smoke-Free Homes’

Members of the research team, who are experts in community health and health education on smoking and health, offered health talks to primary and secondary students in schools between the months of September and November 2010 before the launching of the slogan and drawing competitions. The education talks included (i) basic facts on the ingredients of cigarettes and their damaging effects, (ii) the mechanism of how active smoking and exposure to second- and third-hand smoke affects the health of students and all members of a family living in the same house, (iii) the benefits of a ‘Smoke-Free Home’ and tips on how to establish such a home, and (iv) the importance of the tactics used by students to relay the message to their parents and other family members. Keeping in mind differences in the ages and maturity levels of the students, the health talks were designed and conducted at different levels and in different formats for primary and secondary school students.

#### Slogan and comic drawing ‘story-telling’ competitions

There were three categories of competition for primary and secondary students: a Chinese slogan, an English slogan, and a comic drawing ‘story-telling’ competition. The competitions were launched with the aim of encouraging the primary and secondary students to illustrate the related health messages and organize the health knowledge into creative written or art forms.

Three experts were invited to serve as honorary judges for our competitions. The expert panel included a visual arts teacher, a professor of Chinese language, and a director of the English Language Centre of the University. All of the winners (1st, 2nd, 3rd place, and 10 merits) in each category of the competitions were offered an award certificate and book coupons. These awards were given to winners at the award ceremony, held at the 2011 Health Fair. A theme song to disseminate the message of ‘smoke-free homes’ was specially written by the judge of Chinese slogan competition, and performed at the opening ceremony.

#### Health fair

The health fair was held at one of the seven major Universities in Hong Kong on a Saturday in late June, at the end of school term to maximize the number of participants at the event. An announcement was made to all schools in Hong Kong, and students were invited to bring their family members. The health fair was free of charge and open to the general public. The winning entries in the slogan and the comic drawing competitions were all displayed in various places during the health fair, for the public to view. Other than a project member and a coordinator, a total of 49 nursing students were recruited from the University to help with the planning and hosting of the games and health check booths, as well as with decorating the booths.

Nurse specialists from the various nursing specialties associations also contributed by setting up their own health and game booths that supported the theme of the health fair. These health booths offered free health checks, including blood pressure and pulse measurements, peak flow rate and carbon monoxide testing; blood glucose and cholesterol measurements; and blood artery scanning.

Smoke cessation specialists were on site in a smoking cessation counselling booth to give advice and answer any questions that participants might have regarding smoking and smoking cessation efforts. Also offered at the booth were visual information such as video clips of ‘I Love Smoke-free Hong Kong 2008’; leaflets entitled ‘Getting Rid of Smoking Sets You Free!’ and ‘For a better environment, say NO to second-hand smoke!’, requested officially from the Tobacco Control Office, Department of Health; and poster boards on the different types of methods to help people quit smoking, including nicotine chewing gum, patches, and inhalers.

Besides the health check and smoking cessation booths, there were game booths designed to promote and raise awareness of ‘smoke-free homes’ and healthy living. Themes such as ‘What is Second-hand and Third-hand Smoke?’, The ‘Effects of Second-hand Smoke’, and the ‘Benefits of Smoke-free Homes’ were used to design the game booths. The winning entries in the Chinese and English slogan competitions, along with the visual arts ‘story-telling’ competitions, were also used in the game booths as tongue twisters or puzzles.

A programme booklet was designed, printed, and distributed to participants during the health fair. The booklet included a brief report on the community-wide SFH project, the winning entries to the slogan and visual arts competitions, and information on the 2011 Health Fair, such as a rundown on the opening ceremony and a list of the health check and game booths.

The programme booklet also included a ‘Passport’ section designed to enable the participants to collect stamps after participating in each game, health check, and smoking cessation booth, and filling out questionnaires. Participants could collect these stamps and exchange them for souvenirs. The souvenirs were stationery designed using the winning entries in each category of the slogan and comic drawing competitions.

### Questionnaires

Two sets of questionnaires were handed out to assess the effects of the education talks and leaflets, and the activities of the health fair, on the commitment of the students and the participants of the health fair to not smoke, and to establish a smoke-free home.

The first structured questionnaire was completed in school by the students after they had received the leaflets and health talks (Additional file 1). This questionnaire was developed to obtain information on the school-aged children’s knowledge about the importance of and how to establish smoke-free homes (SFH), and whether they and their parents support the concept of SFH, pledge to maintain an SFH, and promote SFH to their families and friends.

Another questionnaire, contained only nine questions, was distributed to the participants of the health fair (Additional file 2). It was developed to obtain information on the participants’ knowledge of smoking and passive smoking, their awareness of and confidence in maintaining an SFH, and whether they have pledged to take different actions to support SFH.

The items were adopted from questionnaires employed in a related study [12, 18], taking into account the objectives of this health promotion project, the content of the health education talks, educational leaflet, and activities of the health fair. The questionnaires was developed by the project coordinator, and a smoking counsellor, and validated by two experts in smoking research.

### Data collection method

Students who received the educational leaflets and a brief overview of the content from their teachers were asked to bring the leaflets home and share the information with their parents. One week after these leaflets were distributed, students were given a questionnaire to complete. The completed questionnaires were then collected by a member of the research team.

The other questionnaires were distributed to participants of the health fair, who completed them anonymously.

### Data analysis

The participation in various activities was presented in terms of numbers and percentages. A simple descriptive analysis using a Chi square test was used to compare the agreement with, support for, and commitment to promoting smoke-free homes between the primary and secondary students.

### Ethical considerations

Ethical approval was received from the university’s Human Subjects Ethics Sub-committee before the commencement of the study. Ethical approval was received for the study from the principals of the schools where the educational leaflets and questionnaires were distributed. The questionnaires that were distributed at the health fair to participants of the fair were completed anonymously. Those who completed the questionnaires and returned them to the researchers were considered to have given their implied consent to participate in the study.

## Results

A total of 1103 promotional packages were sent to all schools in Hong Kong at the beginning of the school year in early September 2010. Around 550 students received health education on SFH in the schools. A total of 38 schools (12 secondary and 26 primary schools) requested SFH educational leaflets, and 6000 were distributed to their students.

A total of 4561 students from 119 schools (71 secondary and 48 primary schools) participated in the Chinese and English slogan and/or comic drawing competitions. A total of 1236 programme booklets were distributed at the 2011 Health Fair. Table 1 shows the number of participants in the various activities of the project.

**Table 1:** Estimated number of participants in the activities of the community-wide school health project for the promotion of Smoke-Free Homes

Activity			No. of participants
Health talk attendees at seven schools			550
Educational leaflet distributed to 38 schools (12 secondary and 26 primary schools)			6000
Entries to the slogan and visual arts competitions from 119 schools (71 secondary and 48 primary schools)			4561

	Primary students (n = 2255)	Secondary students (n = 2306)	
Chinese slogan	1342	1866	3208
English slogan	356	220	576
Comic drawing	557	220	777
Health fair participants	1236		
Total number of participants reached in all activities:			12,347

### Indications of agreement and support from the students for SFH after having received the educational leaflets

A total of 4805 out of the 5500 questionnaires distributed were returned, giving a response rate of 87.4 %. Of the students who returned the questionnaire, 55 % were female and 45 % were male. Slightly over half of them reported that the educational leaflets increased their knowledge (58.3 %) and awareness (55.0 %) of establishing ‘smoke-free homes’. A majority of them pledged to maintain an SFH (87.6 %), well over half (69.6 %) agreed to promote the concept of SFH to their family and friends, and 88.2 % agreed to advise their family and friends not to smoke at home. More of their mothers (80.8 %) than fathers (66.5 %) also pledged to support the practice of maintaining an SFH. More primary students (90.8 %) than secondary students (85.8 %) reported that they were non-smokers and pledged that they would not take up smoking. About 82 % of those who had experimented with smoking pledged to stop the habit (Table 2).

**Table 2:** Indications of agreement and support for Smoke-free Homes (SFH) after having received the educational leaflets (n = 4805*)

	Total (n = 4805*)		Primary (n = 2005)		Secondary (n = 2800)	
	n	(%)	n	(%)	n	(%)
Effects of promotional materials on facts about the establishment of smoke-free homes						
Increase students’ knowledge^**+**^	2799	(58.3)	1332	(66.4)	1467	(52.4)
Increase students’ awareness^**+**^	2644	(55.0)	1290	(64.3)	1354	(48.4)
Students pledged to practice SFH^+^	4208	(87.6)	1838	(91.7)	2370	(84.6)
Students agreed to						
Promote SFH message to family and friends^**+**^	3344	(69.6)	1569	(78.3)	1775	(63.4)
Discuss with family ways to establish SFH^**+**^	2855	(59.4)	1327	(61.7)	1528	(54.6)
Advise family and friends to quit smoking^**+**^	4226	(88.0)	1862	(92.9)	2364	(84.4)
Advise family and friends not to smoke at home^**+**^	4240	(88.2)	1812	(90.4)	2428	(86.7)
Families’ willingness to support SFH						
Fathers^**+**^	3193	(66.5)	1491	(74.4)	1702	(60.8)
Mothers^**+**^	3884	(80.8)	1720	(85.8)	2164	(77.3)
Decisions regarding smoking						
I have not smoked, and I pledge not to smoke in the future^**+**^	4222	(87.9)	1820	(90.8)	2402	(85.8)

	(n = 583)		(n = 185)		(n = 398)	
Ever-smokers						
I have tried smoking, and I pledge to stop starting from today	480	(82.3)	151	(81.6)	329	(82.7)

* Missing data not >3 %

^+^All with *p* ≤ 0.0001 in the Chi square test

### Report from health fair participants regarding the establishment of an SFH

A total of 300 copies of the second questionnaire were distributed to the participants. About 50 copies were randomly distributed to participants six times throughout the duration of the health fair, at hourly intervals from 11 a.m. to 4 p.m. A total of 201 questionnaires were collected, giving a response rate of 67 %.

About three-fourths of the participants at the health fair reported that the activities at the health fair increased their knowledge of smoking and second- and third-hand smoke (77.1–77.6 %), their awareness of the importance of establishing an SFH (80.1 %), and their confidence in maintaining an SFH (78.6 %) (Table 3).

**Table 3:** Health fair participants’ report on their knowledge of smoking and commitment to establishing a SFH (n = 201)

		n	(%)			
Gender						
Male		61	(30.3)			
Female		130	(64.7)			
Age (mean = 24.4)						
19 or younger		51	(25.4)			
20–34		57	(28.4)			
35–49		40	(19.9)			
50–64		32	(15.9)			
65 or older		18	(9.0)			

	Total		Significant increase		Increase	
	n	(%)	n	(%)	n	(%)
Knowledge of smoking and passive smoking						
Effect of smoking on health	156	(77.6)	61	(30.3)	95	(47.3)
How second-hand smoke exposure affects health	155	(77.1)	64	(31.8)	91	(45.3)
How third-hand smoke exposure affects health	143	(71.1)	64	(31.8)	79	(39.3)
Knowledge and practice of SFH						
Awareness of establishing SFH	161	(80.1)	73	(36.3)	88	(43.8)
Confidence in practicing SFH	158	(78.6)	78	(38.8)	80	(39.8)

			n	(%)		
Among those who do not smoke (n = 162)						
Never smoked, and pledge not to smoke in the future	150	(92.6)				
Among those who smoked (n = 39)						
Smoke, and pledge to take action to quit			31	(79.5)		
Smoke, and pledge not to smoke at home			33	(84.6)		
Participants agreed to						
Pledge to support SFH			184	(91.5)		
Promote SFH to family and friends			176	(87.6)		
Advise family not to smoke at home			167	(83.1)		
Advise family to quit smoking			171	(85.1)		
Discuss with family the ways to establish SFH			158	(78.6)		
Place a no-smoking sign at home			140	(69.7)		
Ask smokers to dispose of lit cigarettes before entering the home	154	(76.6)				
Inform visitors of your SFH policy			157	(78.1)		
Achievements made by families						
Established an SFH policy			30	(14.9)		
Placed a no-smoking sign at home			33	(16.4)		
Had smoking visitors dispose of lit cigarettes before entering the home	32	(15.9)				
Informed visitors of their SFH policy			26	(12.9)		

In addition, the smokers pledged to take action to quit smoking (79.5 %), and to not smoke at home (84.6 %). The majority of the participants (91.5 %) pledged to support SFH, and as much as 69.7–87.6 % agreed to promote SFH to their families and friends, advise their family members to quit, place a ‘No Smoking’ sign at home, and ask visitors not to smoke in their homes. About 12.9–16.4 % reported already having carried out some of the above actions to establish an SFH (Table 3).

## Discussion

This community-wide SFH school health project met its objectives and made an impact in disseminating the message of the benefits of Smoke-Free Homes to about 12,300 school-aged children and an uncountable number of their family members and home visitors. Students received first-hand information on the benefits of smoke-free homes through health talks, educational leaflets, slogans, and visual arts competitions, and the 2011 Health Fair. After participating in the project’s various activities, the participants indicated that their awareness of and confidence in maintaining an SFH had also increased, and many pledged to establish and maintain Smoke-Free Homes.

The results from the project’s questionnaires indicated that the project increased the school-aged students’ awareness of and confidence in maintaining Smoke-Free Homes. The fact that the majority of the participants pledged to establish and maintain Smoke-Free Homes was encouraging. Through the activities in the health fair, students, together with their family members, and other participants also pledged to take action to support the establishment of an SFH, and some have already taken action.

The health promotion effects of this SFH project go beyond reaching families with smokers, and extend to those households without smokers. The household without smokers could have benefited from this health promotional project in that members of such households can promote the message of SFH to members of their other extended family and friends, ask visitors not to smoke in their homes, and eventually contribute to the establishment of a community norm of smoke-free homes.

## Limitations

Although this is the first community-wide school-based health promotion project to promote smoke-free homes, there are limitations to this study. First, while the response rate of the primary and secondary students who completed the questionnaires after receiving the educational leaflet was satisfactory (87.4 %, 4805 out of 5500), only 67 % (201 out of 300 questionnaires distributed) of those participants at the Health Fair who took a questionnaire completed it, amounting to just 16.3 % (201 out of 1236) of all participants at the Health Fair. This limitation may have caused biased outcomes, as those who were willing to complete the questionnaire may be people who are more likely to support smoke-free homes. One should be cautious in interpreting the results of this project.

Another limitation of this health promotion project is that no baseline data were collected. There was also no control group against which to compare the effects. Post-activity outcomes using a questionnaire were on a ‘self-reported increase in the knowledge, awareness, and agreement to support and establish an SFH, and on agreeing to advise’. These are only indicative of the perceptions of the participants, and not necessarily of the effects of the health promotion activities.

Another limitation of this health promotion project is that it measured only the short-term self-reported agreement with, support for, and commitment to the concept of maintaining smoke-free homes, but not the actual effort and actions that the participants/respondents would put into carrying out this health promotive practice or the long-term beneficial outcomes of the project.

However, some of the above limitations are features of community health promotion activities in that the intention was not to withhold a health promotion intervention from the ‘control’ group, and the long-term impacts and changes in community norms and behaviours are often not measurable.

## Conclusion and recommendations

Public health efforts of this kind should be continued to reach the general public in Hong Kong. This project benefited not only the school-aged children, but also their families and friends. Most school-based programmes have been initiated to reduce the incidence of smoking by secondary students [21, 22], and most studies on restrictions on smoking at home have been conducted to examine its association with changes in smoking behaviour [23, 24]. No studies have been found of community-wide school-based programmes similar to this health promotion project on promoting smoke-free homes.

The effects of this project may not be measurable at this stage, but at the very least, it indirectly sent students the message that smoking is an unpleasant behaviour, not a social norm, and damaging to the living of a healthy life. This project may have indirectly decreased the risk of school-aged children becoming the victims of second- and third-hand smoke, and decreased their chances of becoming smokers in the future, in turn improving the quality of life of families and the population of Hong Kong.

Besides a policy to ban smoking in public places in the effort to reduce the exposure of non-smokers to passive smoking, community and public health professionals should also target children who are at a high risk of exposure if a parent smokes at home. An overall strategy for reducing children’s exposure should combine counselling in clinics and offices for smokers, health education on the hazards of smoking and smoking exposure to all populations in the community, public regulations, policies, and taxes [25], as well as the promotion of smoke-free homes! A community-wide health promotion project should aim to reach the public to promote a social norm of not smoking, establish the norm of smoke-free homes, reduce the exposure of adolescents to second-hand smoke, and reduce the number of adolescents who pick up the habit of smoking [22].

It is also recommended that future studies be conducted to examine the effects of school-based health promotion projects on the smoking behaviours of adolescents, or on the smoking practices of family members who smoke.

## Additional files

10.1186/s13104-015-1555-4 Questionnaires for students on smoke-free homes.
10.1186/s13104-015-1555-4 2011 Smoke-free Home Health fair Questionnaire.
