# Supplementary material for: A community-wide school health project for the promotion of smoke-free homes
Source: BMC Res Notes. 2015 Nov 26;8:724. doi: 10.1186/s13104-015-1555-4 (PMC4662028; doi:10.1186/s13104-015-1555-4)
Supplement: Supplementary file 1 — 10.1186/s13104-015-1555-4 Questionnaires for students on smoke-free homes. [file 13104_2015_1555_MOESM1_ESM.pdf]

Questionnaires for students on smoke-free homes

Remark: Smoke-Free Home (SFH) refers to family's agreement on taking action to support or maintain SFH. This means everyone is not allowed to smoke at home so as to protect family from the hazards of second-hand smoke.

Please select the most suitable answer and 「√」 the box

		Yes	No
1.	Have you read the educational leaflets related to SFH of this project?	❑	❑
**2.**	Did the educational leaflets increase your knowledge of SFH?<br>(If you have not read the materials, please select 'No')	❑	❑
**3.**	Did the educational leaflets increase your awareness of SFH?<br>(If you have not read the materials, please select 'No')	❑	❑
4.	Are you interested in participating in SFH activities?		
	■ Health talks	❑	❑
	■ Slogan competitions	❑	❑
	■ Visual arts 'story-telling' competitions	❑	❑
	■ Health fair	❑	❑
**5.**	I pledge to practice SFH.	❑	❑
**6.**	Are you and your families willing to practice and support SFH?		
	■ myself	❑	❑
	■ Father	❑	❑
	■ Mother	❑	❑
	■ Other family members	❑	❑
**7.**	To support SFH, I will practice the followings:		
	I have never smoked, and I pledge not to smoke in the future	❑	❑
	If I have tried smoking,		
	■ I pledge to stop smoking starting today	❑	❑
	■ I pledge not to smoke at home	❑	❑
	If my family and friends are smokers,		
	■ I advise them to quit smoking	❑	❑
	■ I advise them not to smoke at home	❑	❑
	I will discuss with family members on ways to establish SFH	❑	❑
	I will place a 'no-smoking' sign at home	❑	❑
	I will ask smokers to dispose of lit cigarettes before entering the home	❑	❑
	I will promote SFH message to family and friends	❑	❑

8. Your age:
9. Gender: ❑ Male ❑ Female
10. Education level: ❑ primary school ❑ secondary school

End
