# Supplementary material for: A community-wide school health project for the promotion of smoke-free homes
Source: BMC Res Notes. 2015 Nov 26;8:724. doi: 10.1186/s13104-015-1555-4 (PMC4662028; doi:10.1186/s13104-015-1555-4)
Supplement: Supplementary file 2 — 10.1186/s13104-015-1555-4 2011 Smoke-free Home Health fair Questionnaire. [file 13104_2015_1555_MOESM2_ESM.pdf]

2011 Smoke-Free Home Health Fair Questionnaire

Remark: Smoke-Free Home (SFH) refers to family's agreement on taking action to support or maintain SFH. This means everyone is not allowed to smoke at home so as to protect family from the hazards of second-hand smoke.

Please select the most suitable answer and 「√」 the box

		Yes	No
1	Have you participated in health talk of SFH or read the project materials related to SFH (including leaflets, posters or website)?	❑	❑
2	Have you participated in the following SFH Health Fair activities:		
	• Health check	❑	❑
	• Game booths (E.g. harmful effects of active smoking, second-hand and third-hand smoke)	❑	❑
	• Health talks on SFH	❑	❑

3. Comparing with the time before taking part in the 2011 SFH Health fair, do you change the following behaviours now? Please 「√」 the suitable box.	not increased	A little increased	Somewhat increased	Significant increased
a. Your knowledge of the effect of smoking on health	❑	❑	❑	❑
b. Your knowledge of how second-hand smoke exposure affects health	❑	❑	❑	❑
c. Your knowledge of how third-hand smoke exposure affects health	❑	❑	❑	❑
d. Your awareness of the importance of establishing SFH	❑	❑	❑	❑
e. Your confidence in practicing SPF increases	❑	❑	❑	❑

4. To support SFH, I will practice the following:	Yes	No
a. I have never smoked, and pledge not to smoke in the future	❑	❑
b. I do smoke, and pledge to take action to quit	❑	❑
c. I do smoke, and pledge not to smoke at home	❑	❑

5 To support SFH, please indicate if you agree to practice the followings, or if you have already taken these actions:	Have already taken this action	Yes	No
d. I will support SFH	❑	❑	❑
e. I will promote SFH to family and friends	❑	❑	❑
f. I will advise my family not to smoke at home	❑	❑	❑
g. I will advise my family to quit smoking	❑	❑	❑
h. I will discuss with family the ways to establish SFH	❑	❑	❑
i. I will place a no-smoking sign at home	❑	❑	❑
j. I will ask smokers to dispose of lit cigarettes before entering the home	❑	❑	❑
k. I will inform visitors of my SFH policy	❑	❑	❑

7. Gender: ❑ Male ❑ Female

8. Age ❑ 11 – 19 ❑ 20 – 34 ❑ 35 – 49 ❑ 50 – 64 ❑ ≥ 65
